# Eds. Hom. Physician

**Published:** 1889-08

**Authors:** 


Eds. Hom. Physician: Hahnemann gave us Sulphide of Calcium; Villers, Mercurius cyanatus; most of us use Ars. iod., Stibium arsen., Ammon. brom., Lapis alb., and many more combinations. Dr. Lorbacher speaks in their favor, considering them *au fait*, and E. M. Hale goes one better, and recommends double remedies, according to Lutze, especially as physicians of our school constantly order many combinations. I know that some, who pride themselves on their high potencies, use them in the CM, etc., and declare that thus neither alternation nor succession takes place, but that they give a unit. Please inform us on those points, whether it shall be considered progressive Homœopathy or otherwise. S. L.

